# Overview of Space-Capable Global Navigation Satellite Systems Receivers: Heritage, Status and the Trend towards Miniaturization

**DOI:** 10.3390/s23177648

**Published:** 2023-09-04

**Authors:** Eberhard Gill, Jade Morton, Penina Axelrad, Dennis M. Akos, Marianna Centrella, Stefano Speretta

**Affiliations:** 1 Department of Space Engineering, Faculty of Aerospace Engineering, Delft University of Technology, 2629 HS Delft, The Netherlands; m.centrella@tudelft.nl (M.C.); s.speretta@tudelft.nl (S.S.); 2 Ann & H.J. Smead Department of Aerospace Engineering Sciences, University of Colorado Boulder, Bolder, CO 80303, USA; jade.morton@colorado.edu (J.M.); penina.axelrad@colorado.edu (P.A.); dma@colorado.edu (D.M.A.)

**Keywords:** GNSS, space receivers, space missions, COTS, miniaturization

## Abstract

Spaceborne Global Navigation Satellite Systems (GNSS) receivers have become ubiquitous sensors for spacecraft navigation, especially in Low Earth Orbits (LEOs), often also supporting science endeavors or as acting dedicated science payloads. Due to the large number of space-capable GNSS receiver models available, spacecraft designers, as well as scientists, may find it difficult to have or gain an overview of suitable state-of-the-art models for their purposes and constraints. Based on a literature review that included more than 90 different receiver models, this paper aims to provide an overview of space-capable GNSS receivers that have a heritage in space missions. It analyses trends from the collected data and provides an outlook on miniaturized GNSS receiver models, which have a high potential of being used in future space missions.

## 1. Introduction

The benefits of using the Global Navigation System (GPS) to track user satellites have been explored since 1982, when the first GPS receiver, the single-frequency GPSPAC (GPS receiver and processor package designed at JHU/APL, with NASA as the co-sponsor, built by Magnavox) was flown on the Landsat-4 satellite [1,2]. At this time, long before GPS’ full operational capability and with only five active GPS satellites in space, it was recognized that receivers in LEO could not only successfully track GPS satellites but also that this technology had the potential to reduce or avoid the dependency on costly ground-based tracking and provide precise onboard timing. In 1992, the operational use of GPS-based precise orbit determination (POD) was first established when the GPSDR (GPS Demonstration Receiver) dual-frequency receiver (developed by Motorola Inc. under contract by JPL), also known as Monarch, was flown onboard the TOPEX/Poseidon mission [3,4] to directly support scientific altimetry requirements. All subsequent altimetry and gravimetry missions have carried one or more geodetic-quality GNSS receivers for POD purposes. A new dedicated science capability for atmospheric sensing made possible by GPS in LEO was realized by the GPS/MET experiment on board MicroLab-1 in 1995, which was the first to collect GPS-based spaceborne radio occultation (GNSS-RO) measurements [5]. The first spaceborne GNSS-Reflectometry (GNSS-R) measurements were made in 2000 onboard the space shuttle by the SIR-C instrument at a relatively low altitude of 208 km [6].

In terms of technology, spaceborne receivers were developed that supported the increasing number of frequencies and signals of a single GNSS [7]. In addition, efforts to integrate other GNSS constellations, i.e., GLONASS, Galileo, BeiDou, QZSS and NavIC, have been realized for many modern receivers [8]. The characteristics of the GPS, GLONASS, Galileo, and BeiDou systems in terms of their signal-in-space range error (SISRE) for LEO satellites have been analyzed in [9]. Additionally, the era of space-capable Commercial Off-the-Shelf (COTS) receivers started, pushed by the University of Surrey and realized by Surrey Satellite Technology Ltd. (SSTL), United Kingdom [10]. However, using COTS GNSS equipment onboard satellites requires extensive testing and qualification efforts, as shown by way of example for radiation testing [11], which included not only single-frequency but also geodetic-grade, dual-frequency receivers.

The first survey of spaceborne GPS receivers was published as early as 1992 and included 18 receivers with their Size, Weight and Power (SWaP) characteristics [12]. In [13], an overview of GPS receivers and associated missions is provided as well, including a roadmap of future GPS receiver technology developments and applications, such as onboard autonomy and formation flying. An overview and trends in space-capable GPS receivers are also presented in [14]. More recent overviews of space-capable GNSS receivers can be found in [15,16]. However, as the market for these receivers is rather dynamic, these overviews do not fully reflect the current status. In addition, they are focused primarily on high-end receivers for Precise Orbit Determination (POD) or for science applications. In particular, they do not cover the recent developments in terms of the further miniaturization of GNSS receivers developed, e.g., for the Internet Of Things (IoT) and mobile phone markets, with the potential to be modified and used in space. Online resources concerning spaceborne GNSS receivers include [17] which, however, only focuses on missions before 2005. The company satsearch B.V. provides an online directory of satellite products, mostly tailored to small satellites, including GNSS receivers [18]. However, this directory is by far not complete and does not provide a comprehensive overview and analysis.

We restrict our overview to those GNSS receivers that are specifically applicable to user spacecraft navigation. Thus, receivers that are designed for launchers and sounding rockets, such as the ACC-G3IR-LV or Navika-251-HD of the Indian company Accord Software & Systems Private Limited, are not fully characterized in this article. Similarly, GNSS receivers that have been designed primarily for science applications, such as GNSS Radio Occultations (GNSS-RO) and GNSS Reflectometry (GNSS-R), have not been considered. Examples of GNSS-RO receivers and their associated missions comprise GOLPE on SAC-C, GPS/MET on OrbView, GRAS on MetOp A to C, ROSA on OceanSat-2, IGOR on Formosat-3/COSMIC and TriG on Formosat-7/COSMIC-2. Examples of GNSS-R receivers and their associated missions comprise PYCARO on ^3^Cat-2 [19] and the SSTL SGR-ReSI onboard the TechDemoSat. A collection of these earlier GNSS-RO and GNSS-R receivers can be found in Table 6.4 and Table 6.14 of [20].

When selecting an appropriate GNSS receiver for any space mission, the mission requirements and constraints govern any informed decision. They may vary tremendously, depending on the specific mission. In Table 1, a number of key criteria in the selection of GNSS receivers are listed together with reasons why these are relevant.

The overall performance of receivers in real space missions, in particular the accuracy that can be achieved, depends not only on the receiver itself but also on the spacecraft design, such as the GNSS antenna and its orientation, and the usage of the receiver’s data, such as which data types are used and how they are processed. A good overview of aspects impacting the performance of GNSS receivers is provided in [16] and other related chapters. Even if receivers have already been flown on other satellites, extensive functional testing, including the use of a GNSS Signal Simulator (GSS), is an absolute necessity on top of rigorous integration testing. In addition, and, in particular for COTS receivers, environmental testing is mandatory and can require considerable effort [21]. It typically comprises pyrotechnic shock, random vibration, thermal–vacuum and electromagnetic interference (EMI), electromagnetic compatibility (EMC) (e.g., according to a tailored Mil-Std-461F standard) and radiation testing.

The objective of this paper is to provide an overview of the heritage, status and outlook of space-capable GNSS receivers. This overview is intended to support mission designers and engineers with data that can help them select a space-capable GNSS that can adhere to the needs and constraints of a space mission. Additionally, it can help scientists who are interested in GNSS-based spaceborne data to identify possible candidate receivers for their applications. As no current overview of space-capable receivers exists, this paper will provide essential information for both user groups.

Section 2 provides an overview of space-capable GNSS receivers, their heritage and current status, with a focus on SWaP characteristics and the constellation and frequency information used. Section 3 presents an overview of future candidate space receivers as well as trends in the current receiver development with a focus on miniaturization and special receivers, such as snapshot receivers, which provide interesting promises. Associated challenges in these areas, related to the usage of such receivers in space, are also discussed.

## 2. Heritage and Status

This section presents space-capable GNSS receiver models and their characteristics. It has been compiled from an extensive literature study and also used online resources, either as introduced in Section 1 or the data sheets of individual receiver suppliers. The qualification as being space-capable is either based on the fact that the receivers have already been flown on specific space missions or the fact that it is based on an explicit statement of the manufacturer that the particular receiver is suitable for use in space. Receivers specifically designed for launchers or sounding rockets have not been included in the overview.

The receiver characteristics are mostly taken from publicly available information, such as scientific publications or data sheets. The type of collected data comprises five regimes: model, supplier and country of origin, radio frequency (RF) and tracking characteristics, including number of supported antennas, SWaP values, radiation tolerance, as well as sample heritage space missions, in which those receivers have been used. Performance characteristics, such as TTFF and the accuracy of measurements or position fixes, have not been included, as they may depend on the specific circumstances under which the receivers were tested.

### 2.1. Overview and Statistics

A total of 57 space-capable GNSS receivers have been identified. These include receivers that are currently available on the market as well as receivers that may no longer be available. Since the availability of receivers may change rapidly and depends on companies’ business plans, availability was not included in the overview. In contrast, a few receivers, especially used in missions where agencies are involved, show a long period over which they are considered. An example is Javad’s TRE-G3T receiver, which will be a core part of the ACES experiment onboard the ISS [22].

**Figure 1:**
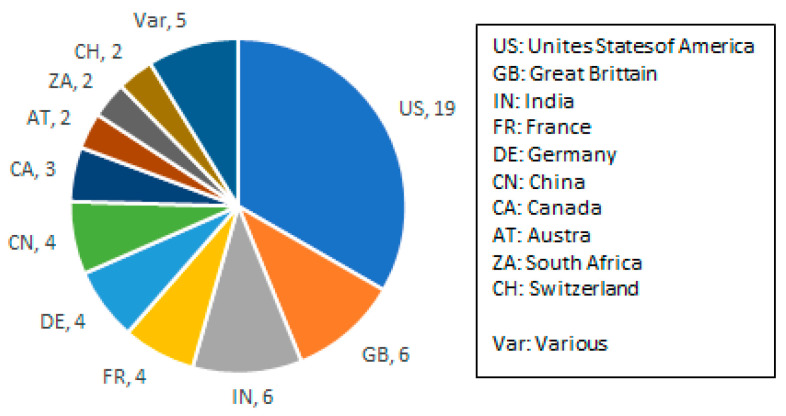
Suppliers of space-capable GNSS receivers for navigation purposes (country codes according to ISO 3166-1 standard).

Figure 1 shows the distribution of the number of space-capable GNSS receiver models per country. The number of GNSS receiver models per country is explicitly shown if more than one. Five countries have developed one model. Those have been summarized under “Var” in Figure 1. It is obvious that the US, by far, dominates this overview, while other countries, such as India, entered the market not too long ago with various models. This is of key importance as space-grade receivers might be export-controlled and difficult to source in many countries.

### 2.2. GNSS Navigation Receivers

Table A1, Appendix A, outlines 57 space-capable GNSS receivers used for navigation purposes.

### 2.3. Analysis

**Figure 2:**
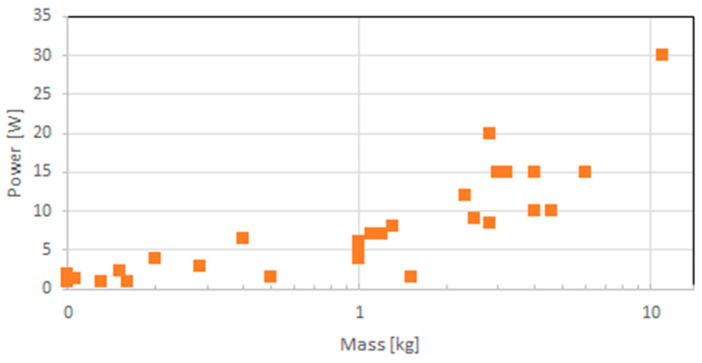
Mass and maximum power distribution of space-capable GNSS receivers.

It is instructive to analyze two aspects of space-capable GNSS receivers: one based on their SWaP values and one on their architecture. Figure 2 shows the mass and maximum power distribution of those receivers, below 14 kg and 35 W, respectively. Two receivers, TriG and Trig-RO of JPL and Moog Broad Reach, have power consumptions higher than this threshold, with 55 and 60 W, respectively, and thus, are not shown in the figure. Similarly, two receivers have masses of more than 14 kg, the GPSDR (Monarch) of JPL with 28 kg and the SAAB GRAS/GPSRS receiver with 30 kg and are, therefore, also not shown in Figure 1. Obviously, a mostly linear relation of mass and power (P [W] = 2.045 + 2.871 m/kg, R^2^ = 0.79) can be observed with high scatter. Here, R^2^ is a measure of goodness of fit, which is the proportion of variance in the dependent variable that is explained by the model. Certainly, it is more important to recognize that the 34 receivers with masses below 1 kg are mostly newer receivers, stressing the trend towards miniaturization, which is further discussed in Section 3.

**Figure 3:**
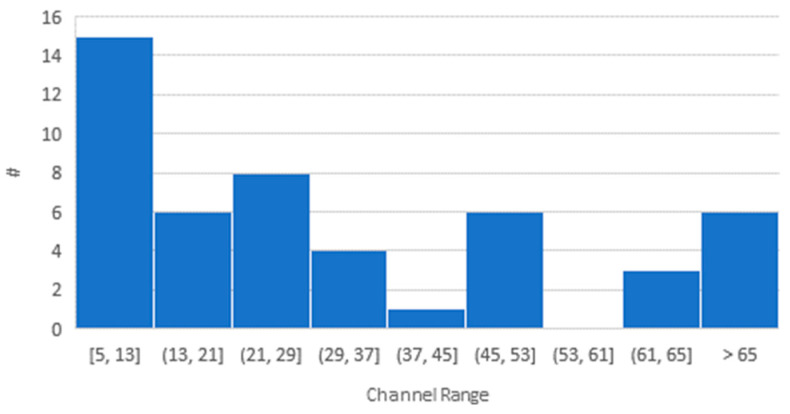
Histogram of number (#) of channels of space-capable GNSS receivers. The bin width has been set to 8 and the overflow bin has been set to 65.

Further, the histogram of the maximum number of channels over the entire data set of space-capable receivers is shown in Figure 3. It can be seen that receivers with 13 or fewer channels have the highest count, with 15 (28%). A closer look reveals that many receivers have channel numbers of multiples of i*12, with i = {1, 2, 4}, which may show the relation of the receiver architectures with the nominal GPS constellation size of 24 satellites.

## 3. Outlook and Trends

Table A1 and Figure 2 show a clear trend towards the miniaturization of space-capable receivers. While for receivers with a heritage of use in space, there could be made a distinction between robust L1 receivers used for onboard navigation and geodetic-grade dual-frequency receivers for science purposes; this distinction is getting more and more blurred. An example of this trend towards high-quality dual-frequency receivers with small form factors is the Polarx-2 receiver of Septentrio [23], with a mass of less than 0.2 kg.

In addition, the use of COTS receivers has gained more and more relevance for missions that in the past considered the use of custom receivers. While single, large spacecraft of space agencies, like the mini-satellite CHAMP of DLR and NASA, were designed to address scientific objectives, commercial companies are currently providing data to NASA and NOAA to support scientific applications ranging from atmospheric and ionospheric monitoring to observations of the surface properties of the Earth. Recently, several commercial companies have launched and operated small satellites with science payloads for GNSS-RO and GNSS-R applications. Examples include STRATO on Spire Global’s LEMUR [24], CION on GeoOptics CICERO [25], and Pyxis on PlanetIQ’s GNOMES [26] constellations. The receivers on these low-cost, 3U-12U CubeSats process multiple constellations of GNSS signals through delay Doppler maps (DDM) and/or open loop tracking to generate measurements for atmospheric, ionospheric, and Earth surface observations. This is a paradigm change in three key aspects: from agency-driven towards industry-provided approaches, from single to multiple satellite architectures and from large spacecraft (the mini-satellite CHAMP had a launch mass of 500 kg) to highly miniaturized spacecraft (each Lemur satellite has a mass of about 6 kg).

### 3.1. Future Candidate Space Receivers

Based on the trends described above, a list of 41 GNSS receivers has been compiled in Appendix B, Table A2, which have not yet been used in space. This list is by no means complete, however. It originated from literature research on companies that have already produced GNSS receivers with space heritage but provide a larger portfolio of GNSS receivers than the explicit space-capable receivers (e.g., Septentrio, Hexagon|Novatel) or by companies that have developed receivers for the mass market and have not yet made a step towards providing space-capable receivers (e.g., u-blox).

In the United States of America prior to 2016, GPS receivers designed to operate at orbital velocities and altitudes were included on the United States Munitions List (USML) and subject to export restrictions under International Traffic in Arms Regulations (ITAR) controlled by the U.S. Department of State. The respective limits were set to a maximum height of 18 km (60,000 ft) and a speed of less than 515 m/s (1000 nm/h). However, the Export Control Initiative [27,28] resulted in a review and modification of the USML such that commercial spaceborne receivers are now covered under the Export Administration Regulations (EARs) process on the Commerce Control List (CCL) Item 9A5115.x controlled by the U.S. Department of Commerce [28]. So, while some export review for spaceborne receivers is required, the process is less restrictive and onerous under the current framework. The European Commission applies in its European regulation a speed limit of 600 m/s [29].

**Figure 4:**
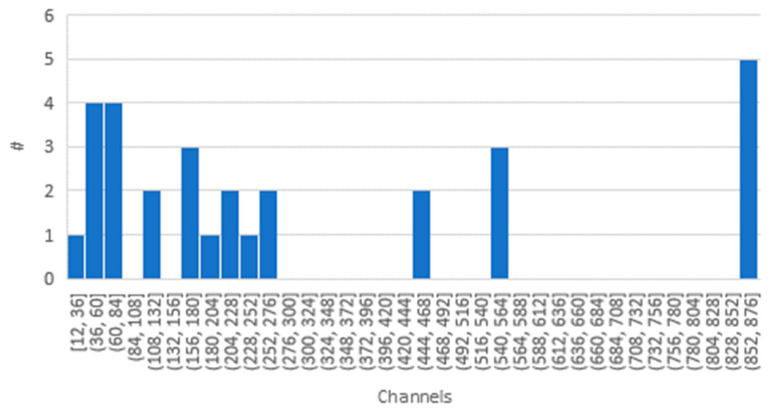
Histogram of number (#) of tracking channels of GNSS receivers that could possibly be used in space. The bin width was set to 24.

Apart from these legal and regulatory aspects, receivers not yet flown in space would need to undergo an extensive testing program, as outlined in Section 1. The development of the GNSS, with its increasing number of constellations, augmentation systems, frequencies and signals, as well as the innovations in receiver technology, such as software-defined radios, are clearly visible in Figure 4. Here, while still almost 25% of the receivers have less than 100 tracking channels, some receivers offer up to 874 tracking channels.

### 3.2. Miniaturization

**Figure 5:**
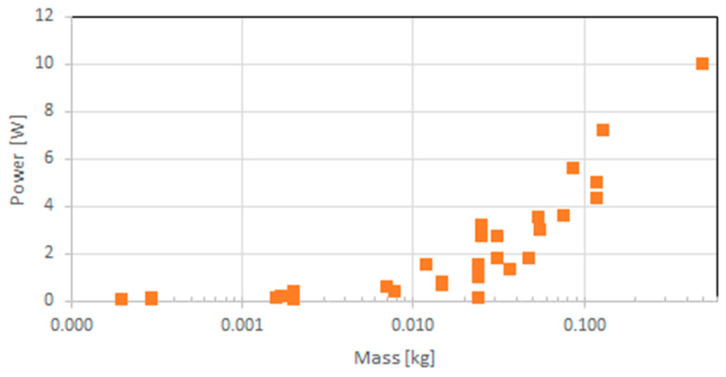
Mass and maximum power distribution of GNSS receivers that could possibly be used in space.

The miniaturization of GNSS receivers is not a new trend. Already in 1998, an advanced GPS receiver for spacecraft was announced under the title “GPS On A Chip”, which led to the BlackJack receiver, with a mass of 3.2 kg [30]. The trend toward miniaturization was also described in [14]. Based on the dataset presented in Table A2, the SWaP distribution of those receivers is shown in Figure 5. It is obvious that even well below the one-kilogram limit, the vast majority of receivers have a mass below 0.1 kg and require less than 10 W of power. Similarly, as for the space-capable receivers, a linear relation between mass and power can be observed (P [W] = 0.938 + 23.041 m/kg, R^2^ = 0.74).

**Figure 6:**
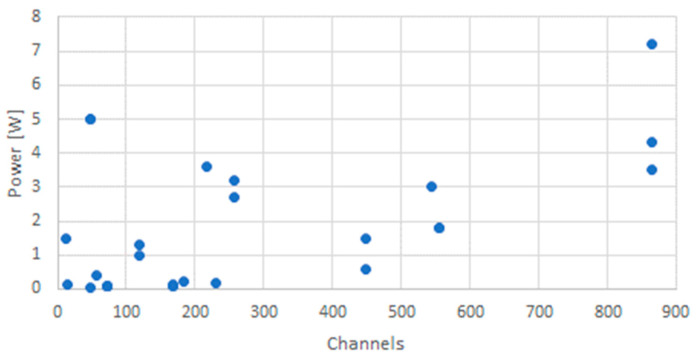
Maximum power consumption and number of channels of GNSS receivers that could possibly be used in space.

For very small satellites, the aspects of power usage may become more relevant than size and mass. Thus, the dataset has been analyzed in terms of power usage and number of available channels. The distribution is shown in Figure 6.

Obviously, there is only a vague relationship between maximum power and the number of channels. This is an interesting phenomenon. A possible explanation could be the different technologies used by the receivers as well as the quality of the data given by the suppliers, which may not follow a standardized approach on how to arrive at the power values given in the data sheets. Other factors include the duty cycle and date rate of the onboard processing. Average power may be a better indicator of the receiver power consumption.

Since energy consumption is a key driving factor, particularly for very small satellites, GNSS receivers requiring very little power may become relevant for space applications in the future. One special type is a GNSS snapshot receiver. In contrast to traditional GNSS receivers, snapshot receivers sleep for most of the time and wake up at defined intervals to record short snapshots, e.g., milliseconds, of GNSS signals. These receivers then digitize the raw signals and store them locally, while the processing of these signals and the estimation algorithms is performed across separate processors. An example of such a snapshot receiver is the SnapperGPS [31], with an estimated form factor of 30 × 30 × 10 mm^3^, an estimated mass of 0.003 kg and a power consumption of 12.6 mAh per year. The use of a snapshot receiver in space is not new. In fact, as part of the “Falcon Gold” experiment, a hosted payload comprising a NAVSYS TIDGET sensor attached to a Centaur upper stage collected in November 1997 data from signal acquisition of GPS satellites [32] in a snapshot mode. In February 1998, the microGPS receiver started to collect snapshots for navigation purposes onboard the SNOE mini-satellite [33]. GNSS snapshot receivers are used for orbit determination on Planet’s Dove satellites [34]. The use of these receivers for positioning is also considered in the framework of Internet of Things (IoT) applications [35]. The use of such snapshot receivers onboard satellites to improve the empirical density models of the upper thermosphere for enhanced Space Situational Awareness (SSA) has recently been proposed [36].

## 4. Conclusions

Based on an extensive literature study, an overview is presented of 57 space-capable GNSS receivers and their characteristics to help spacecraft designers and scientists alike with informed decisions on the receiver selection. The receivers’ SWaP characteristics are discussed, showing a clear trend of miniaturization. Additionally, their architecture is characterized based on their number of tracking channels. In addition, a total of 41 GNSS receivers are presented along with their characteristics, which may be suitable candidates for future usage onboard satellites. There are three areas that still inhibit such usage: commercially, the low numbers of receivers in space are not attractive to suppliers to establish commercial usage; technically, they need to undergo a thorough test and validation program to qualify them for the harsh space environment; and third, legal and regulatory constraints would have to be overcome, which could technically be solved by removing the velocity limits imposed by dual-use regulations and by adapting the Doppler tracking windows.

## Figures and Tables

**Table 1:** Key criteria for GNSS receiver selection and their relevance.

Area	Criteria	Relevance
Performance	Position accuracy	Key criteria without postprocessing
Performance	Velocity accuracy	May impact prediction performance
Performance	Time-To-First-Fix (TTFF)	Time-critical and robust operations
Architecture	Frequencies	High-accuracy science applications
Architecture	Channels	Quality and robust operations
Architecture	Antennas	Science or ADCS ^1^ requirements
Data and I/O ^2^	Raw data	Data postprocessing
Data and I/O	PPS ^3^	Payload or onboard time tagging
Data and I/O	Update rate	Temporal resolution
Operations	Initialization	Effort of operations
Physical	Form factor	Physical spacecraft constraints
Physical	Mass	Subsystem budgets and launch cost
Physical	Power	EPS ^4^ subsystem budget
Physical	Radiation tolerance	Robustness and longevity
Programmatic	Cost	Test effort and mission cost
Programmatic	Legal and regulatory	Specific to country of origin

^1^ Attitude Determination and Control System. ^2^ Input/Output. ^3^ Pulse Per Second ^4^ Electric Power System.

## Data Availability

Not applicable.

## Appendix A

**Table A1:** Space-capable GNSS navigation receivers and their characteristics.

Model Supplier (Country)	Channels Signals	Ant.	Power [W]	Mass [gr]	Size [mm]	TID [krad]	Missions	Reference
Viceroy General Dynamics (US)	12–18 GPS L1 C/A	1–2	7.0	1100	N/A	15	MSTI-3, Seastar, MIR, Orbview	[16]
Viceroy-4 General Dynamics (US)	12 GPS L1 C/A	1–2	7.0	1100	152 × 132 × 43	N/A	MIR, KompSat-1, GOES	[37]
Sentinel M-Code General Dynamics (US)	64 GPS L1 & L2, C/A Code, P(Y), M-Code, L2C optional	2	<9	2500	180 × 60 × 60	100	Classified	[38]
Explorer General Dynamics (US)	12 GPS L1 C/A	1–2	7.0	1200	160 × 142 × 43	N/A	N/A	[39]
GPSDR (Monarch) JPL/Motorola (US)	6 GPS L1, C/A, L2, P	1	29.0	28,000	N/A	N/A	Topex/Poseidon 1992	[3]
GEM-S Rockwell Collins (US)	5 GPS L1 C/A P	1	6.5	400	140 × 150 × 15	N/A	BIRD	[40]
TurboRogue NASA/JPL (US)	N/A	N/A	N/A	N/A	N/A	N/A	MicroLab-1, Oersted	[5]
BlackJack (TRSR-2) NASA/JPL (US)	48 GPS L1, P1, P2	4	15.0	3200	N/A	N/A	CHAMP	[41]
IGOR Broad Reach Eng. (US)	16 × 3 GPS L1 C/A, L1/L2 P(Y)	4	10.0	4600	N/A	20	Formosat-3/COSMIC, TerraSAR-X, TanDEM-X	[16]
TriG JPL, MOOG Broad Reach (US)	24 × 2 GPS/GLO L1/L2, (GAL E1/E5a)	4	55.0	6000	190 × 220 × 120	N/A	Formosat-7/COSMIC-2	[16]
TriG RO MOOG Broad Reach (US)	16 L1 L2 L5 Lx	4–16	60.0	5200	190 × 220 × 120	N/A	Sentinal 6A	[42]
TriG POD MOOG Broad Reach (US)	16 GPS L1 L2 L5 Lx	4	20.0	2800	190 × 140 × 120	N/A	OTB-1 (Orbital Test Bed-1)	[42]
Navigator MOOG Broad Reach (US)	12 GPS L1 C/A	2	12.0	2300	190 × 240 × 80	>100	MMS, Shuttle-HSM-4	[42]
Pyxis POD MOOG Broad Reach (US)	12 GPS L1, L1G, E2	4	20.0	2800	190 × 140 × 120	N/A	N/A	[15]
TRE-G3T Javad (US)	216 GPS L1, L2, L5, Galileo E1, E5a, E5b, AltBOC, E6, Beidou B1, B2	N/A	3.60	77	80 × 100	N/A	ACES Experiment on ISS	[43]
TR-2G Javad (US)	216 GPS L1, Galileo E1, BeiDou E1, QZSS B1, SBAS L1, KFK WAAS/EGNOS	1	N/A	21	57 × 88 × 12	N/A	N/A	[44]
Stratos Spire (US)	N/A GPS L1, L2	N/A	~4	~200	N/A	N/A	Lemur	[45]
GPSRM 1 Pumpkin Inc. (US)	24 GPS L1/L2/L2C and GLONASS L1/L2	1	1.3	106	96 × 90 × 12	N/A	N/A	[46]
OEMV-1G Hexagon | NovAtel (CA)	14 GPS L1	N/A	1.0	21	46 × 71 × 13	N/A	RAX, CanX 4/5	[47]
OEM4-G2L Hexagon | NovAtel (CA)	24 GPS L1, L2, L3, L5, Galileo E1, E5a, E5b, AltBOC, Beidou B1, B2 (all tbc)	N/A	1.6	56	100 × 60 × 16	N/A	CASSIOPE, CanX-2	[48]
OEM4-719 Hexagon | NovAtel (CA)	555 GPS L1, L2, L5, Galileo E1, Beidou B1 (all but E6,B3)	N/A	1.8	31	46 × 71 × 11	N/A	Bobcat-1	[49]
PODRIX RUAG (AT)	18 × 2 GPS L1/L2/L5, Galileo E1/E5a	1	15.0	3000	N/A	50	(SWARM, Sentinel-3)	[15]
GPS POD RUAG (AT)	8 × 3 GPS L1 C/A, L1/L2 P(Y)	1	8.5	2800	N/A	>20	SWARM, Sentinel, ICEsat-2	[16]
PolaRx2 Septentrio (BE)	48 GPS L1, L2	N/A	N/A	190	180 × 100 × 15	9	Tet-1	[23]
NaviLEO SpacePNT (CH)	< = 48 GPS L1 C/A L5I/Q Galileo E1b E5a E6	1–2	8.0	1300	234 × 121 × 66	N/A	N/A	[50]
PODRIX Beyond Gravity (CH)	2 × 18 GPS L1, L2, Galileo E1, E5a	2	15.0	3000	280 × 240 × 81		Sentinel-6A	[51]
Celeste Spacemanic, s.r.o (CZ)	N/A N/A	1	0.1	25	67 × 42 × 7		N/A	[52]
LION Airbus (DE)	36 GPS L1/L2/L5, Galileo E1/E5a	1–4	15.0	6000	N/A	50	SARah, CSO, Metop-5G	[16]
Mosaic GNSS Airbus (DE)	8 GPS L1 C/A	1	10.0	4000	N/A	10	SARLupe, TerraSAR-X, Aeolus	[16]
Phoenix-S DLR (DE)	12 GPS L1 C/A	1	0.9	100	N/A	15	PROBA-2 & -V, PRISMA, TET	[16]
GPS-110 BST (DE)	N/A N/A	2	3.0	285	91 × 84 × 41	N/A	Kent Ridge-1	[53]
TopStar 3000 Thales-Alenia (FR)	12–16 GPS L1 C/A	1–4	1.5	1500	N/A	>30	Demeter, Kompsat-2	[16]
Lagrange Thales-Alenia (FR)	12 × 3 GPS L1 C/A, L1/L2 P(Y)	1	30.0	5200	N/A	20	Radarsat-2, COSMO-Skymed, GOCE	[16]
Tensor Thales-Alenia (FR)	9 GPS L1 C/A	1–4	15.0	4000	N/A	100	Globalstar, SAC-C, ATV	[15]
GNSS S/W Rcv. Syrlinks (FR)	9 GPS L1 C/A, Galileo E1	1	5.0	1000	N/A	10	Taranis	[16]
N-SPHERE SAFRAN (FR)	N/A N/A	N/A	N/A	N/A	N/A	15	GOMX-5 Gomspace A/S	[54]
GNSS-701 AAC ClydeSpace (GB)	120 GPS L1, Beidou B1, Galileo E1	1	1.0	160	94 × 56 × 26	10	N/A	[55]
SGR-05U SSTL (GB)	12 GPS L1 C/A	1 (tbc)	0.8	45	70 × 45 × 10	>11	N/A	[56]
SGR-10 SSTL (GB)	24 GPS L1 C/A	2	5.5	1000	N/A	10	Tsinghua-1, BILSAT, DART	[16]
SGR-20 SSTL (GB)	24 GPS L1 C/A	4	6.0	1000	160 × 180 × 50 (est.)	N/A	TopSat, Uo-Sat 12, OCO-3, Proba-1	[57]
SGR-Axio SSTL (GB)	24 GPS L1 C/A, Option G2, E1, L2C	1–4	4.0	1000	160 × 180 × 50	<5	N/A	[58]
SGR-Ligo SSTL (GB)	24 GPS L1 C/A, Option G1, E1	1–2	<0.5	90	92 × 87 × 12	>5	N/A	[59]
GRAS/GPSOS SAAB (SE)	12 GPS C/A, P1/2	3	30.0	30,000	N/A	N/A	METOP	[15]
NGPS-03-422 NewSpace Systems (ZA)	12 GPS L1	1	1.0	130	96 × 96 × 18	10	N/A	[60]
NGPS-01-422 NewSpace Systems (ZA)	12 GPS L1	1	1.5	500	155 × 76 × 34	10	N/A	[60]
Orion B16-C Navspark (TW)	230 Dual-frequency GPS/GLONASS/Galileo/Beidou/QZSS	act.	0.21	1.6	12 × 16 × 3	N/A	N/A	[61]
GS50 GranStal (CN)	48 GPS L1, GLONASS Lq, Beidou B1	1	0.8	30	51 × 17	N/A	N/A	[62]
GSD700 GranStal (CN)	440 GPS: L1, L2C/L2P, L5GLONASS L1, L2, BeiDou: B1, B2, B3, Galileo E1, E5a, E5b, SBAS L1 C/	1	1.7	45	100 × 60 × 9	N/A	N/A	[63]
COSGNSS COSATS Co., Ltd. (CN)	N/A BDS B1/B2, GPS L1/L2	1	2.4	150	56 × 54 × 11	N/A	N/A	[64]
J150 Beidoustar (CN)	GPS L1/L2, BDS B1/B2/B3, GLONASS G1/G2	N/A	2.0	100	41 × 71 × 13	N/A	Q-Sat	N/A
ACC-GPS-NANO-DR Accord (IN)	2 × 32 GPS L1	1	0.5	45	65 × 75 × 20	20	N/A	[65]
ACC-GPS-NANO-NR Accord (IN)	32 GPS L1	1	0.5	45	65 × 75 × 20	20	N/A	[66]
ACC-GPS-NavIC-NANO Accord (IN)	25 GPS L1	1	1.35	<45	50 × 70 × 14	20	N/A	[67]
ACC-GPS-NANO Accord (IN)	32 GPS L1, SBAS GAGAN	1	0.5	45	64 × 75 × 1.5	20	N/A	[68]
GPS module WARPSPACE (JP)	167 GPS L1 C/A, GLONASS L1	1	0.15	3	24 × 24 × 5	N/A	N/A	[69]

Act. = active. N/A = Not available; SBAS = Satellite-based Augmentation Systems; GAGAN = GPS-aided GEO augmented navigation [70].

## Appendix B

**Table A2:** Candidates for future space-capable receivers and their characteristics.

Model Supplier (Country)	Channels Signals	Ant.	Power [W]	Mass [gr]	Size [mm]	Constraints *	Reference
TR-G2T Javad (US)	216 GPS: L1/L2/L2C/L5 Galileo: E1/E5A SBAS	1	1.60	34	57 × 66	NLS	[71]
TR-3N Javad (US)	864 GPS: C/A, L1C (P+D), P1, P2, L2C (L+M), L5(I+Q) GLONASS: C/A, L2C, P1, P2, L3 (I+Q) Galileo: E1 (B+C), E5A (I+Q), E5B (I+Q), AltBoc BeiDou: B1, B1-2, B1C(P+D), B5A (I+Q), B2, B5B (I+Q) QZSS: C/A, L1C (P+D), L2C (L+M), L5 (I+Q), SAIF SBAS: L1, L5 IRNSS L5	1	3.50	54	57 × 88 × 12	NLS	[72]
TR-3S Javad (US)	874 GPS: C/A, L1C (P+D) including TMBOC (6,1,4/33), P1, P2, L2C (L+M), L5 (I+Q) GLONASS: C/A, P1, P2, L2C, L3 (I+Q) Galileo: E1 (B+C) including CBOC (6,1,1/11), E5A (I+Q), E5B (I+Q), AltBoc, E6 (B+C) Beidou: B1, B1C (P+D) including TMBOC (6,1,4/33), B2B (I+Q), B2, B2A (I+Q), AltBoc, B3 QZSS: C/A, L1C (P+D) including TMBOC (6,1,4/33), L2C (L+M), L5 (I+Q), L6 (L61/L62), L1S, L1Sb, L5SL1, L5 (P+D) SBAS: L1, L5 (P+D) IRNSS: L5, S	1	N/A	30	66 × 57 × 11	NLS	[73]
TR-2S Javad (US)	874 GPS: C/A, L1C (P+D) including TMBOC (6,1,4/33), P1, P2, L2C (L+M), L5 (I+Q) GLONASS: E1 (B+C) including CBOC (6,1,1/11), E5A (I+Q), E5B (I+Q), AltBoc, E6 (B+C) Galileo: E1 (B+C) including CBOC (6,1,1/11), E5A (I+Q), E5B (I+Q), AltBoc, E6 (B+C) BeiDou: B1, B1C (P+D) including TMBOC (6,1,4/33), B2B (I+Q), B2, B2A (I+Q), AltBoc, B3 QZSS: C/A, L1C (P+D); TMBOC (6,1,4/33), L2C (L+M), L5 (I+Q), L6 (L61/L62), L1S, L1Sb, L5S SBAS: L1, L5 (P+D), IRNSS: L5	1	N/A	20	55 × 40 × 11	NLS	[74]
TRE-3S Javad (US)	N/A GPS: C/A, L1C (P+D) including TMBOC (6,1,4/33), P1, P2, L2C (L+M), L5 (I+Q) GLONASS: C/A, P1, P2, L2C, L3 (I+Q) Galileo: E1 (B+C) including CBOC (6,1,1/11), E5A (I+Q), E5B (I+Q), AltBoc, E6 (B+C) QZSS: C/A, L1C (P+D) including TMBOC (6,1,4/33), L2C (L+M), L5 (I+Q), L6 (L61/L62), L1S, L1Sb, L5S BeiDou: B1, B1C (P+D) including TMBOC (6,1,4/33), B2B (I+Q), B2, B2A (I+Q), AltBoc, B3 IRNSS: L5, S SBAS: L1, L5 (P+D)	1	3.7–5.6	87	80 × 100	NLS	[75]
TRE-DUO Javad (US)	864 GPS: C/A, L1C (P+D), P1, P2, L2C (L+M), L5(I+Q) GLONASS: C/A, L2C, P1, P2, L3 (I+Q) Galileo: E1 (B+C), E5A (I+Q), E5B (I+Q), AltBoc BeiDou: B1, B1-2, B1C(P+D), B5A (I+Q), B2, B5B (I+Q) QZSS: C/A, L1C (P+D), L2C (L+M), L5 (I+Q), SAIF SBAS: L1, L5 IRNSS L5	2	4.30	120	100 × 120	NLS	[76]
TRE-Quattro Javad (US)	864 GPS: C/A, P1, P2, L2C (L+M), L1C (I+Q) Galileo: E1 (B+C) GLONASS: C/A, P1, P2, L2C QZSS: C/A, L2C (L+M), L1C (I+Q), SAIF BeiDou: B1,B1R, L1C (I+Q) SBAS: L1	4	7.20	130	100 × 120	NLS	[77]
Quattro-R Javad (US)	216 GPS: L1, L2, L2C	4	5.20	130	100 × 120	NLS	[78]
OEM615 Hexagon|NovAtel (CA)	120 GPS: L1, L2, L2C GLONASS: L1, L2 BeiDou: B1 Galileo: E1 SBAS QZSS	N/A	1.00	<24	46 × 71 × 11	VL	[43]
OEM628 Hexagon|NovAtel (CA)	120 GPS: L1, L2, L2C, L5 GLONASS: L1, L2 BeidDou: B1, B2 Galileo: E1, E5a, E5b, AltBOC SBAS QZSS: L1, L2C, L5	N/A	1.30	37	60 × 100	VL	[43]
OEM7500 Hexagon|NovAtel (CA)	N/A GPS: L1 C/A, L1C, L2C, L2P, L5 GLONASS: L1 C/A, L2 C/A, L2P, L3 Galileo: E1, E5a, E5b, AltBOC BeiDou: B1I, B1C, B2I, B2a, B2b QZSS: L1 C/A, L1C, L1S, L2C, L5 NavIC (IRNSS): L5 SBAS L1, L5 L-Band up to 5 channels	N/A	1.50	12	33 × 55 × 4	VL	[79]
OEM7600 Hexagon|NovAtel (CA)	555 GPS: L1 C/A, L1C, L2C, L2P, L5 GLONASS: L1 C/A, L2 C/A, L2P, L3, L5 Galileo: E1, E5 AltBOC, E5a, E5b BeiDou: B1I, B1C, B2I, B2a, B2b QZSS: L1 C/A, L1C, L1S, L2C, L5 NavIC (IRNSS): L5 SBAS: L1, L5 L-Band: up to 5 channels	N/A	1.80	31	35 × 55 × 13	VL	[80]
OEM7700 Hexagon|NovAtel (CA)	555 GPS: L1 C/A, L1C, L2C, L2P, L5 GLONASS: L1 C/A, L2 C/A, L2P, L3, L5 Galileo: E1, E5 AltBOC, E5a, E5b, E6 BeiDou: B1I, B1C, B2I, B2a, B2b, B3I QZSS: L1 C/A, L1C, L1S, L2C, L5, L6 NavIC (IRNSS): L5 SBAS: L1, L5 L-Band: up to 5 channels	N/A	1.80	31	46 × 71 × 8	VL	[81]
OEM7720 Hexagon|NovAtel (CA)	555 GPS: L1 C/A, L1C, L2C, L2P, L5 GLONASS: L1 C/A, L2 C/A, L2P, L3, L5 Galileo: E1, E5 AltBOC, E5a, E5b BeiDou: B1I, B1C, B2I, B2a, B2b QZSS: L1 C/A, L1C, L1S, L2C, L5 NavIC (IRNSS): L5 SBAS: L1, L5 L-Band: up to 5 channels	2	2.70	29	46 × 71 × 8	VL	[82]
OEM729 Hexagon|NovAtel (CA)	555 GPS: L1 C/A, L1C, L2C, L2P, L5 GLONASS: L1 C/A, L2 C/A, L2P, L3, L5 Galileo: E1, E5 AltBOC, E5a, E5b, E6 BeiDou: B1I, B1C, B2I, B2a, B2b, B3I QZSS: L1 C/A, L1C, L1S, L2C, L5, L6 NavIC (IRNSS): L5 SBAS: L1, L5 L-Band: up to 5 channels	1	1.80	48	60 × 100 × 9	VL	[83]
simpleRTK2B lite ArduSimple (AD)	N/A GPS: L1C/A L2C GLONASS: L1OF L2OF Galileo: E1-B/C E5b BeiDou: B1I B2I QZSS: L1C/A L2C SBAS: WAAS, EGNOS, MSAS, GAGAN and SouthPAN	1	0.40	7.8	41 × 28	NLS	[84]
simpleRTK3B pro ArduSimple (AD)	448 GPS: L1C/A L1PY L2C L2P L5 GLONASS: L1CA L2CA L2P L3 CDMA Galileo: E1 E5a E5b E5 AltBloc E6 BeiDou: B1I B1C B2a B2I B3 QZSS: L1C/A L2C L5 Navic: L5 SBAS: WAAS, EGNOS, MSAS, GAGAN, SDCM (L1 L5)	1	1.50	24	69 × 53	NLS	[85]
AsteRx4 Septentrio (BE)	544 GPS: L1, L2, L5 GLONASS: L1, L2, L3 Galileo: E1, E5ab, AltBoc, E6 BeiDou: B1, B2, B3 IRNSS: L5 QZSS: L1, L2, L5 Galileo, Beidou, IRNSS, E6/B3 and AltBoc are optional features	N/A	3.00	55	61 × 82	VL	[43]
mosaic-X5 Septentrio (BE)	448 GPS: L1C/A, L1PY, L2C, L2P, L5 GLONASS: L1CA, L2CA, L2P, L3 CDMA Beidou: B1I, B1C, B2a, B2b, B2I, B3 Galileo: E1, E5a, E5b, E5 AltBoc, E6 QZSS: L1C/A, L1 C/B, L2C, L5 Navic: L5 SBAS: Egnos, WAAS, GAGAN, MSAS SDCM (L1, L5) On module L-band	N/A	0.60	7	31 × 31 × 4	VL	[86]
PolaRx2e@ Septentrio (BE)	48 L1, L2 SBAS: EGNOS, WAAS	3	5.00	120	160 × 100 × 13	VL, HL	[87]
PolaRx2eh Septentrio (BE)	48 L1, L2 SBAS: EGNOS, WAAS	2	5.00	120	160 × 100 × 13	VL, HL	[87]
ZED-F9P-04B u-blox (CH)	184 GPS: L1C/A L2C GLONASS: L1OF L2OF Galileo: E1B/C E5b BeiDou: B1I B2I QZSS: L1C/A L1S L2C SBAS: L1C/A	N/A	0.21	2.0 (est.)	17 × 22 × 2.4	CoCom	[88]
MAX-M8Q u-blox (CH)	72 GPS: L1 C/A QZSS: L1 C/A SAIF GLONASS: L10F BeiDou: B1I Galileo: E1B/C SBAS: L1 C/A: WAAS, EGNOS, MSAS, GAGAN	N/A	0.07	2.0 (est.)	9.7 × 10.1 × 2.5	CoCom	[89]
MAX-M8W u-blox (CH)	72 GPS: L1 C/A QZSS: L1 C/A SAIF GLONASS: L10F BeiDou: B1I Galileo: E1B/C SBAS: L1 C/A: WAAS, EGNOS, MSAS, GAGAN	N/A	0.07	2.0 (est.)	9.7 × 10.1 × 2.5	CoCom	[90]
MAX-M8C u-blox (CH)	72 GPS: L1 C/A QZSS: L1 C/A SAIF GLONASS: L10F BeiDou: B1I Galileo: E1B/C SBAS: L1 C/A: WAAS, EGNOS, MSAS, GAGAN	N/A	0.07	2.0 (est.)	9.7 × 10.1 × 2.5	CoCom	[91]
NEO-M8Q-01A u-blox (CH)	72 GPS: L1 C/A QZSS: L1 C/A GLONASS: L10F BeiDou: B1I Galileo: E1B/C SBAS: L1 C/A: WAAS, EGNOS, MSAS, GAGAN	N/A	0.07	2.0 (est.)	12.2 × 16.0 × 2.4	CoCom	[92]
piNAV-NG SkyFox Labs (CZ)	15 GPS: L1 C/A	1	0.13	24	71.1 × 45.7 × 11	NLS	[93]
Q20 QinetiQ (GB)	12 C/A L1	1 (act.)	0.1–1.522	N/A	35 × 50 × 5	VL	[94]
NTL102.SMT NTLab (LT)	N/A GPS: L1, L5 NavIC: L5, S-band and SBAS L1	2	<0.65	<15	30.5 × 25.5 × 4.2	NLS	[95]
NTL103.SMT NTLab (LT)	N/A GPS: L1, L2 GLONASS: L1, L2 NavIC: L5,S-band and SBAS L1	2	0.19–0.8	<15	38 × 25.5 × 3.5	NLS	[96]
NTL104.SMT NTLab (LT)	256+128 GPS: L1, L2, L5 GLONASS: L1, L2 Galileo: E1, E5a, E5b BeiDou: B1, B2 NavIC (IRNSS) L5, S-band and SBAS L1	2	3.20	<25	71 × 46 × 10	NLS	[97]
NTL106.SMT NTLab (LT)	256 GPS: L1, L2/L5 GLONASS: L1, L2 Galileo: E1, E5a/E5b BeiDou: B1, B2 NavIC (IRNSS) L5, S-band and SBAS L1	1	2.70	25	71 × 46 × 10	NLS	[98]
TESEO-LIV3F STMicroelect- ronics N.V. (NL)	N/A GPS: L1C/A GLONASS: L1OF BeiDou: B1 Galileo: E1B/C SBAS: L1C/A QZSS: L1C/A	1	0.08	N/A	9.7 × 10	NLS	[99]
STA8089G STMicroelect- ronics N.V. (NL)	48 GPS, Galileo, GLONASS, BeiDou and QZSS	1	0.04	N/A	7 × 7 × 1.0	NLS	[99]
S1216F8-GI3 SkyTraQ (TW)	56 GPS: L1 GLONASS: L1 Gagan: L1 NavIC L5, (not GPS L5)	1	0.40	2	12 × 16	NLS	[100]
S1216F8-GL SkyTraQ (TW)	167 GPS: L1 C/A GLONASS: L1 C/A	1	0.13	1.6	12.2 × 16	NLS	[101]
PX1122C SkyTraQ (TW)	230 GPS: L1/L2C Galileo E1/E5b Beidou: B1I/B2I QZSS: L1/L2C	1	0.17	1.7	12 × 16	NLS	[102]
Venus816 SkyTraQ (TW)	N/A GPS: L1 GLONASS: L1/L2 QZSS, SBAS Capable	2	0.07	0.3 (est.)	5 × 5	VL, HL	[103]
Venus828F SkyTraQ (TW)	N/A GPS: L1 Beidou: B1 QZSS, SBAS	N/A	0.07	0.2	7 × 7 × 1.4	VL, HL	[104]
Venus838FLPx SkyTraQ (TW)	167 L1, B1	N/A	0.10	0.3	10 × 10 × 1.3	VL, HL	[105]
NanoSense GPS Kit GomSpace A/S (DK)	167 GPS: L1	N/A	1.80	31	46 × 72 × 11	CoCom removed	[106]
SoftSpot Syntony (FR)	555 GPS: L1 C/A; L1C; L2C; L5 Galileo: E1-OS; E5a; E5b; E6-CS GLONASS: G1 & G2 BEIDOU: B1 & B2 SBAS: WAAS; EGNOS; MSAS Military codes, IRNSS, GBAS, DGPS	N/A	10.00	500	131 × 106 × 25	N/A	[107]

* NLS = No Limits Specified; VL = Velocity Limits; HL = Height Limits. Act. = active; CoCom = Coordinating Committee for Multilateral Export Controls; DGPS = Differential GPS; EGNOS = European Geostationary Navigation Overlay Service; GAGAN = GPS-Aided Geo-Augmented Navigation [70]; GBAS = Ground-Based Augmentation System; IRNSS = Indian Regional Navigation Satellite System; MSAS = Multi-functional Satellite Augmentation System; SAIF = Submeter-class Augmentation with Integrity Function; SouthPAN = Southern Positioning Augmentation Network; SBAS = Satellite-based Augmentation Systems; SDCM = System for Differential Corrections and Monitoring; WAAS = Wide-Area Augmentation System.
